# “To Anticipate”: Neoadjuvant Therapy in Melanoma with a Focus on Predictive Biomarkers

**DOI:** 10.3390/cancers12071941

**Published:** 2020-07-17

**Authors:** Mattia Garutti, Silvia Buriolla, Elisa Bertoli, Maria Grazia Vitale, Ernesto Rossi, Giovanni Schinzari, Alessandro Marco Minisini, Fabio Puglisi

**Affiliations:** 1 Dipartimento di Oncologia Medica, Centro di Riferimento Oncologico di Aviano (CRO) IRCCS, 33081 Aviano, Italy; silvia.buriolla@cro.it (S.B.); elisa.bertoli@cro.it (E.B.); fabio.puglisi@cro.it (F.P.); 2 Department of Medicine (DAME), University of Udine, 33100 Udine, Italy; marygracevitale89@gmail.com (M.G.V.); alessandro.minisini@asuiud.sanita.fvg.it (A.M.M.); 3 Medical Oncology, Fondazione Policlinico Universitario A. Gemelli IRCCS, 00168 Rome, Italy; ernestorossi.rm@gmail.com (E.R.); giovanni.schinzari@gmail.com (G.S.); 4 Medical Oncology, Università Cattolica del Sacro Cuore, 00168 Rome, Italy; 5 Dipartimento di Oncologia, Azienda Sanitaria Universitaria del Friuli Centrale, 33100 Udine, Italy

**Keywords:** melanoma, neoadjuvant, adjuvant, immunotherapy, BRAF, MEK

## Abstract

Despite surgical resection and adjuvant therapies, stage III melanomas still have a substantial risk of relapse. Neoadjuvant therapy is an emerging strategy that might offer superior efficacy compared to adjuvant therapy. Moreover, neoadjuvant therapy has some virtual advantages: it might allow for less demolitive surgery, permit the in vivo evaluation of drug efficacy, help tailor adjuvant treatments, and play a crucial role in innovative translational research. Herein, we review the available literature to explore the scientific background behind the neoadjuvant approach. We also discuss published clinical trials with a focus on predictive biomarkers and ongoing studies. Finally, we outline a possible framework for future neoadjuvant clinical trial development based on the International Neoadjuvant Melanoma Consortium guidelines.

## 1. Introduction

Anti-BRAF/MEK agents and immune checkpoint inhibitors (ICIs), which have proven their efficacy in metastatic melanoma patients, have been tested as an adjuvant therapy for resected stage III high-risk melanoma to reduce the risk of recurrence and improve distant metastases-free survival (DMFS) and overall survival (OS) [1,2,3,4,5,6]. The efficacy of such agents has been confirmed, and they are currently used as adjuvant treatments. However, more than 30% of the patients with stage III disease relapse within 24 months [7].

Despite surgical resection, high-risk stage II (e.g., stage IIC) patients can have even worse survival than some stage III patients [8]. Interferon-alpha (INF-alpha) can be offered to these patients as an adjuvant treatment [9]. This treatment, however, offers only a modest clinical benefit at the cost of remarkable toxicity [9]. Indeed, clinical trials are also ongoing to evaluate ICIs in stage II melanoma patients (KEYNOTE-716, CheckMate 76K).

An emerging issue is evaluating effective treatments for melanoma through neoadjuvant therapy. This approach offers some additional benefits compared to adjuvant treatments alone [10,11,12,13].

First, the tumor shrinkage after induction therapy can translate into less demolitive surgery with inferior morbidity and an increased chance to be radical [10,11,12,13].

Second, neoadjuvant therapy offers an in vivo test for the efficacy of the drugs, thus allowing a tailored adjuvant approach. For example, an ongoing clinical trial PRADO is evaluating the possibility to de-escalate or escalate systemic adjuvant therapies and the relevance of complete dissection in patients who achieved a complete pathologic response (pCR) [14,15]. Notably, pCR is related to improved OS and DMFS in other solid tumors, and the FDA approved the use of pCR as the primary endpoint in neoadjuvant trials in breast cancer [16,17,18,19]. For melanoma, a pooled analysis of six neoadjuvant studies demonstrated an association between pCR and relapse-free survival (RFS). In particular, the 12-month RFS rate was 95% vs. 62% (*p* = 0.001) (for patients who achieved pCR and for patients without pCR, respectively) [20].

Third, an analysis of modifications to the tumor and its microenvironment after neoadjuvant therapy might provide useful insights for translational research. In particular, it will be important to identify the mechanisms of resistance and predictive biomarkers, such as the gene expression signature, tumor-infiltrating T-cells, and tumor mutational burden, to better select the patients who could most benefit from different types of therapies.

Fourth, it has been hypothesized that, for melanoma, the neoadjuvant approach could confer a possible advantage in survival compared to adjuvant strategies [21,22] with the early treatment of micrometastatic disease. Indeed, preclinical data showed more significant activity of ICIs in the neoadjuvant setting compared to the adjuvant one, because of greater T-cell expansion and a reduced impairment of T-cell functions [21].

All these advantages should be weighted against the risk of cancer progression during neoadjuvant treatment. However, this risk seems to be low (see below). 

We reviewed all the available studies on neoadjuvant treatments for stage III melanoma, with a specific focus on the predictive factors of response (Table 1).

## 2. First Experiences in the Neoadjuvant Setting

Before the introduction of ICIs and anti-BRAF/MEK agents, chemotherapy was the standard of care for melanoma patients.

Studies conducted on advanced melanoma patients reported a 10–20% response with Dacarbazine and up to 40% with a combination of cytotoxic drugs without a substantial survival benefit [23,24].

To improve the outcome of metastatic melanoma patients, trials with biochemotherapy, including Interleukin-2 (IL-2) and/or IFN-alfa, in association with chemotherapy have been conducted. A meta-analysis by Ives reported a response rate (RR) of up to 50% with biochemotherapy without a survival improvement [25].

Considering the RR, biochemotherapy was also tested in a neoadjuvant setting. Buzaid described a 48% RR with neoadjuvant biochemotherapy, including 10% complete responses (CR) [26], similar to metastatic melanoma [25].

Forty-eight patients with stage III melanoma were treated with Cisplatin, Dacarbazine, and Vinblastine, in association with IL-2 and IFN-alfa [27]. The response rate was 38.9% with 11.1% pCR. After a 31-month follow-up, 64.6% of the patients were free of disease progression, but the study reported severe toxicity with this schedule [27].

Lewis reported the results of another phase II study, enrolling 92 patients with stage III melanoma, with either clinical lymph node or sentinel lymph node positivity, and treated with biochemotherapy [28]. The RFS and the OS after a median follow-up of 40.4 months were 64% and 78%, respectively, and a high toxicity rate was confirmed [28].

A retrospective study considering 153 patients treated in a single institution with biochemotherapy in a neoadjuvant setting reported toxicity in 46% of the patients. These adverse events were associated with a worse survival, suggesting that the inability to tolerate neoadjuvant biochemotherapy could be a negative prognostic factor [29].

In a study by Koyanagi, circulating tumor cells (CTCs), during neoadjuvant biochemotherapy, were associated with a worse prognosis. Therefore, CTC monitoring could help predict patient outcomes [30].

Temozolomide was also tested in melanoma patient candidates for surgical resection. The RR was 16%, similar to that in a metastatic setting [31]. Moschos explored the role of a high dose of interferon-a2b in patients with palpable lymph nodes, observing 55% clinical responses. The results obtained in this study suggest an immunomodulatory effect of high dose interferon [32].

The trials with IL-2/IFN and chemotherapy as neoadjuvant agents did not demonstrate a survival advantage, but they represent the basis for a promising neoadjuvant strategy in the treatment of stage III melanoma patients.

## 3. Neoadjuvant Immunotherapy 

Preclinically, it has been shown that neoadjuvant immunotherapy confers a better prognosis compared to the adjuvant approach [21]. This effect could be due to its capability to increase the number of tumor-specific CD8^+^ T-cell clones. This specific immune system activation is essentially mediated by the recognition of neoantigens expressed selectively by cancer cells [33]. From this perspective, the presence of a major tumor mass is fundamental to amplify the tumor-specific T-cell response, while increasing the expansion of such cells in the peripheral blood. Accordingly, Amaria and Huang demonstrated greater T-cell expansion in neoadjuvant immunotherapy compared to adjuvant therapy [22,34]. 

Considering the positive results of anti-PDL-1 and anti-CTLA4 combination therapy in advanced-stage melanoma [35] and adjuvant settings [36], several clinical trials were designed to develop immunotherapy in a neoadjuvant setting. 

### 3.1. Ipilimumab Single Agent 

Neoadjuvant ipilimumab was tested by Tarhini et al. [37] in locally/regionally advanced melanoma, to evaluate the safety and toxicity of two courses of ipilimumab (10 mg/kg) every three weeks before surgery, followed by two additional adjuvant doses. This study, for translational purposes, aimed to evaluate the role of ipilimumab in modulating the expression of cellular immunosuppression markers in the tumor microenvironment and in the blood. Thirty-five patients were enrolled, and 33 were evaluated for efficacy. Eight patients (24%) had disease progression, and 5 (15%) had minimal residual disease at pathological assessment. According to the primary endpoint, no grade 4–5 toxicity was reported; the adverse events observed included grade 3 diarrhea/colitis, hepatitis, rash, and elevated lipase, with an incidence rate ranging from 3% to 14% [37]. 

Moreover, the cytokine and chemokine profiles were evaluated, and the IL-7 blood levels showed a significant correlation with grade 3 colitis, supporting the connection between immune-related toxicity and ipilimumab’s mechanism of action [38]. In addition, TGF-β1 and IL-10 levels were associated with a lower and higher risk of recurrence, respectively, which is consistent with previous data [39]. The assessment of blood samples collected six weeks after the start of ipilimumab revealed a transient increase in circulating T-regulatory cells (Treg) and a decrease in circulating myeloid-derived suppressor cells (MDSC), which are both associated with improved RFS [37]. In the overall population, the median PFS was 11 months with a median follow up of at least 16 months. Moreover, no difference in circulating Treg levels was observed between relapse-free and relapsed patients at three, six, and nine months [37].

The updated long-term results [40] reported a positive impact of lower baseline levels of circulating Treg on RFS, while higher levels of CD39^+^ and FoxP3^+^ Treg were observed in the relapsed patients. Similarly, lower Treg in the tumor was related to clinical benefits. Within the tumor, Ipilimumab induced several modifications, such as increased infiltration by activated T-cells (CD69^+^, CD3/4^+^, and CD3/8^+^), the induction of memory cells (CD45RO^+^), and decreased MDSC, all leading to an improved 1-y RFS [40]. These findings support the immunomodulatory role of the CTLA-4 blockade concerning the host response. 

### 3.2. Pembrolizumab Single Agent 

Huang et al. [41] tested neoadjuvant pembrolizumab monotherapy in stage IIIB-C and resectable stage IV melanoma patients. This phase I study aimed to evaluate the efficacy and safety of a single dose of pembrolizumab (200 mg IV) followed by radical surgery three weeks later, with adjuvant pembrolizumab for up to one year. Twenty-nine patients were enrolled and treated using the pre-planned surgical timing, due to the acceptable treatment toxicity and absence of grade 4–5 adverse events. The pathological response was assessed in 27 patients with an overall RR of 30% (8/27; complete in 5/27, 19%; major complete response 3/27, 11%) [41]. 

According to previous data [22,34,42,43], the pathological response has a relevant prognostic value: patients with pCR and near-pCR were all disease-free at a median follow up of 25 months (a 2 y disease-free survival (DFS) of 100%), whereas patients without a significant pathological response had a higher risk of recurrence (10/19 patients recurred, 7 with metastatic disease). In the overall population, the 1-y DFS was 63%, and the median DFS was not reached [41]. 

Besides pCR, the radiographic response (available in six patients), decreased viable tumor (<50%), and increased tumor-infiltrating lymphocytes (TILs) were all associated with improved DFS [41]. In particular, the percentage of patients with brisk TILs (defined as lymphocytes diffusely infiltrating the invasive component of the tumor) increased after treatment, with a significant improvement in 1-y RFS (89% vs. 27% in non-brisk TIL patients) [44]. Paired pre- and post-treatment blood samples were collected and analyzed for translational purposes. Seven days after treatment, a robust improvement in Ki67^+^ CD8 T-cells was observed, particularly in cells expressing PD-1, CTLA-4, and markers of T-cell exhaustion, such as CD39 [41]. Intriguingly, Ki67^+^ cells during pre-treatment, day 7, and week 3 presented a similar differentiation phenotype, corroborating the role of the PD-1 blockade in revitalizing a pre-existent subset of T-cells [41].

In the peripheral blood, an increased proliferation of CD8^+^, CD4^+^, and FoxP3^+^ regulatory cells was detected after pembrolizumab; in the tumors, an expansion of CD8^+^ T-cells and T-lymphocytes expressing exhaustion markers (bet and eomesodermin) was observed, featuring a strong association with clinical benefits, consistent with the preclinical models [21].

Notably, an exhausted phenotype is commonly associated with T cell effector-function deterioration and the expression of immunosuppressor receptors [45]. Therefore, the reinvigoration of exhausted T cells, particularly the progenitor subset, could overcome the tumoral immune escape through the differentiation in cytotoxic TILs [46]. By analyzing the potential determinants of anti-PD1 resistance, an increase in PD-L1 and Tregs emerged, which was proportional to CD8 T-cell proliferation, thereby suggesting a concomitant rapid upregulation of the immunoregulatory feedback in patients with poor DFS [41]. 

### 3.3. Anti-PDL-1 and Anti-CTLA-4 Combination Therapy 

In a phase Ib OpACIN trial, 20 patients with clinical stage III melanoma were randomly chosen to receive Ipilimumab 3 mg/kg (IPI3) and Nivolumab 1 mg/kg (NIVO1), as four courses of adjuvant therapy or divided into two neoadjuvant treatments followed by two post-operative courses [22]. Notably, no delay or complications in surgery were reported, even though 90% of patients developed grade 3–4 immune-related adverse events (irAEs) (mostly within the first 12 weeks of treatment) in both arms. At the last update, the 3 y rate was 80% in the neoadjuvant arm compared to 60% in the adjuvant arm [47]. The pathologic response rate (pRR), defined as <50% of viable tumor cells, was 78%. The pathological response was reported in three patients. After a median follow-up of 36.7 months, none of the patients who achieved pCR after neoadjuvant treatment relapsed, thus making pCR an encouraging potential surrogate marker for neoadjuvant ICIs RFS and OS [47]. 

The randomized phase II trial of Amaria et al. compared four doses of neoadjuvant Nivolumab 3 mg/kg (NIVO3), versus up to three doses of combined IPI3+NIVO1 in clinical stage III and oligometastatic stage IV melanoma [34]. The combination arms showed a higher rate of grade ≥3 toxicity (73%), and early disease progression occurred in 2 out of 12 patients in the single-agent Nivolumab arm, precluding surgical resection. For these reasons, the trial was prematurely interrupted. The RR was 25% and 73%, while pCR was achieved in 25% and 45% of patients in the nivolumab monotherapy arm and combination arm, respectively. The only available information on the neoadjuvant nivolumab monotherapy was derived from the control arm of the Amaria study [34]. Neoadjuvant nivolumab might have a higher RR compared to its use in a metastatic setting (CheckMate 067 trial) (45% vs. 25%, respectively), and lower toxicity compared to both advanced (CheckMate 067 trial) and adjuvant therapy (CheckMate 238 trial) (grade 3–4 8% vs. 23% vs. 14%, respectively).

The small numbers of patients enrolled combined with premature interruptions make it difficult to obtain conclusive clinical efficacy data from these two studies [22,34]. However, interesting translational insights were generated. In the OpACIN trial, the extent of the expansion of tumor resident T-cell clones in peripheral blood was superior and broader in the neoadjuvant arm compared to the adjuvant arm. Furthermore, a higher expansion of new T-clones, together with increased T-cell tumor infiltrate, was detected in the non-relapsed versus relapsed patients. Lower levels of CD3 (a marker of T-cell infiltration), β2-microglobulin (reflecting MHC expression), and the PDL-1 molecule in cancer were strongly associated with a relapse, either after neoadjuvant or adjuvant ICI therapy. Moreover, the high/intermediate RNA expression of IFN-γ signature, determined by RNA sequencing, identified patients with a better outcome [22]. This agrees with previous evidence showing that IFN-γ signaling influences T-cell activity and modulates responses to ICIs [22,48]. Moreover, in Amaria et al., the CD8^+^ T-cell tumor infiltrate, PD-L1, and lymphoid markers (granzymes B, CD4/20, PD-1) were higher in the baseline and early-treatment tumor samples of the responders. A greater expansion of peripheral blood tumor-resident T-cell clones was observed in the combination arm, although this did not reflect differences in response [34]. Notably, tumor mutational burden (TMB) was not associated with responses in either study [22,34,48]. 

Given the toxicity of the combination therapy and the poor responses with the risk of progression and inoperability among the patients treated with ICI monotherapy, the OpACIN-neo trial was designed to preserve a high response rate while minimizing the toxicity of combination therapy [49]. In that study, 105 patients were randomly assigned (1:1:1) to two cycles of ipilimumab 3 mg/kg plus nivolumab, 1 mg/kg, once every three weeks (IPI3 + NIVO1); two cycles of ipilimumab 1 mg/kg plus nivolumab, 3 mg/kg, once every three weeks (IPI1 + NIVO3) or two cycles of ipilimumab, 3 mg/kg, once every three weeks, directly followed by two cycles of nivolumab, 3 mg/kg, once every two weeks. Primary endpoints were grade ≥3 irAEs during the first 12 weeks (based on OpaCIN trial results), RR, and pRR. At a median follow-up of 7.7 months, the IPI3 + NIVO3 arm was prematurely closed, due to the high incidence of severe irAEs (five cases of grade 3 colitis, one of whom required a colectomy, and one case of grade 4 polyneuropathy). All treatment discontinuations were caused by the irAEs: 13% in the IPI3 + NIVO1 arm, 17% in the IPI1 + NIVO3 arm, and 31% in the IPI3 + NIVO3 arm [49]. Accordingly, in the Checkmate 511 trial [50], a lower but not significantly different incidence of grade 3/4 irAEs was observed in IPI1 + NIVO3 versus IPI3 + NIVO1 (grade ≥3 20% vs. 40% within 12 weeks from the start of ICI). However, the incidence of irAEs observed in IPI3 + NIVO1 was less than expected, based on the OpACIN trial. An RR was achieved in 63%, 57%, and 35% of patients in the IPI3 + NIVO1, IPI1 + NIVO3, and IPI3 + NIVO3 arms, respectively. The pRR was 77% in the IPI1 + NIVO3 arm, which was also the arm with the highest pCR (57%) [49]. These data confirmed the previous OpACIN observations, in which the radiological response underestimated the pathological response. Thus, this trial met its primary goal in identifying the two courses of the IPI1 + NIVO3 regimen as the best benefit/toxicity schedule [49]. Although immature for analysis, at a median follow-up of 24.6 months after randomization, the median RFS was not reached in any of the arms, and only one (2%) of the patients who experienced a pathologic response relapsed (versus 64.5% of non-responders) [51]. 

In the pooled analysis of these two studies, pCR was observed in 38% of patients and was associated with a better RFS: a 12-month RFS in 100% vs. 72% in patients with or without pCR, respectively (*p* < 0.001) [20]. Taken together, these results highlight that neoadjuvant combination therapy IPI3 + NIVO1 can provide a higher RR, but possibly at the price of greater grade 3/4 toxicity versus the same regimen in the checkmate 067 phase III trial under first-line metastatic settings (73% vs. 58% for RR and 90% vs. 59% for safety, respectively) [35]. However, the two courses of neoadjuvant IPI1 + NIVO3 achieved similar RR results (57%) in reducing toxicity (20%) [50]. 

In ancillary translational studies of the OpaCIN-neo trial, contrary to the results of Amaria et al. [34], PD-L1 expression did not influence the pathologic response rate, and higher plasma levels were observed after the therapy, independently from the response [49,52]. Similar to the Checkmate 238 trial findings, the baseline high IFN-γ signature, determined by a tumor gene expression analysis, and TMB were associated with a higher response to ICIs [36]. Thus, a combination of the IFN-γ signature and TMB at baseline could identify a subgroup of likely responders. The discordant results of these three trials emphasize that the relationship between TMB and the response to ICI is not yet completely understood [53]. 

As suggested in the OpACIN-neo trial, pathologic response is an important marker for RFS, even if its long-term benefits remain to be proven [52]. In a small prospective study, 20% of patients treated with neoadjuvant immunotherapy (both a mono- and combination-regimen) avoided surgical management due to a complete radiological response and remained recurrence-free at a median follow up of 31.8 months [54]. This opens up the possibility for the more conservative management of patients to achieve pCR. In line with Routy et al. [55], the baseline intestinal microbial diversity was significantly lower in non-responders [56]. 

## 4. BRAF/MEK Inhibitors

The use of targeted therapy as a (neo)adjuvant treatment has been reported since 2013 in many retrospective studies and case series [14,15,57]. However, only two studies showed the possible role of targeted therapy (particularly Dabrafenib and Trametinib) in the neoadjuvant landscape. 

Amaria et al. [42] reported the results of the CombiNeo trial, which enrolled 21 patients with stage III or oligometastatic stage IV BRAF V600-mutated melanoma. This trial randomized 1:2 using the standard of care (surgery followed by adjuvant therapy) or eight weeks of neoadjuvant Dabrafenib and Trametinib followed by surgery and continued as an adjuvant treatment for up to a total of 52 weeks. Overall, at a median follow up of 18.6 months, nearly 71% of patients in the experimental arm remained alive without disease progression, whereas all patients in the control arm progressed [42]. 

The NeoCombi is a single-arm, open-label, single-center phase II study that was performed at the Melanoma Institute Australia. This study, patients with IIIB-C melanoma received Dabrafenib and Trametinib for a total of 52 weeks (12 weeks before complete resection and for 40 weeks after surgery). The study enrolled 35 patients, and the median follow-up was 27 months [58]. Nearly 86% of patients achieved a response according to RECIST; in particular, 16 (46%) had a CR, and 14 (40%) had a partial response. Five patients (14%) had stable disease, and no patients progressed. After resection and pathological evaluation, 100% of patients achieved a pathological response: 17 (49%) patients had a pCR, and 18 (51%) had a partial pathological response [58]. These results highlight the high proportion of patients that achieved a pCR with neoadjuvant Dabrafenib plus Trametinib. In terms of safety, the data are in line with previously published works, and no grade 5 issues were reported. Furthermore, during the 12-week neoadjuvant period, no progression was reported, and all patients were able to undergo surgery [58]. This approach could be considered especially for the subset of patients for whom neoadjuvant immunotherapy might not be suitable.

From a translational perspective, the biomarker analyses available from the NeoCombi and CombiNeo trials showed lower phosphorylation of ERK at baseline in patients who achieved pCR and, intriguingly, a higher expression of TIM3 and LAG3 in the TILs of patients who did not achieve a pCR [42,58]. These data suggest the association of ICI with BRAF/MEK inhibitors in increasing the rate of response and RFS.

## 5. BRAF/MEK Inhibitors versus Immunotherapy

The results of the immunotherapy and BRAF/MEK inhibitor trials in neoadjuvant settings suggest some relevant questions regarding their different levels of efficacy. In particular, the pooled analysis of Menzies et al. suggests an increased activity of immunotherapy in comparison to BRAF/MEK inhibitors. The 12-month RFS was improved in patients who received immunotherapy compared to targeted therapy treatment (83% vs. 65%, *p* < 0.001). Notably, the RFS rates at 24 months for those with pCR after immunotherapy treatment were 100% (72% for those without pCR RFS rates). Instead, for those with pCR after targeted treatment, RFS was 78% (compared with 8% for those without pCR) [20].

Another pivotal issue concerns the predictive biomarkers for immunotherapy and BRAF/MEK inhibitors to better select the right treatments for patients. For targeted therapy, remarkable results were observed in a recently published biomarker analysis of an adjuvant COMBI-AD trial, in which patients were randomized to Dabrafenib and Trametinib vs. placebo [59]. High INF-gamma-gene signature expression was shown to be a prognostic factor for prolonged RFS survival among all patients in the trial. Instead, low TMB was associated with a long-term relapse-free survival for Dabrafenib and Trametinib [59]. Since a high TMB was previously associated with responses to immunotherapy [60], this biomarker might be useful in selecting patients for neoadjuvant targeted therapy vs. immunotherapy. However, prospective trials are needed to confirm these hypotheses. 

## 6. Intralesional Therapy

Intralesional treatments represent a promising strategy for melanoma neoadjuvant therapy. This treatment modality could determine a profound tumor response, and potential advantages include the possibility to elicit both a local- and a systemic-immune response while reducing systemic adverse events. The first demonstration of melanoma cell eradication in a neoadjuvant setting employed polylactic acid microspheres (PLAMs) loaded with IL-12 or TNF-a in a B16 melanoma mice model [61]. This preclinical study showed complete tumor eradication and a systemically specific tumor response [61].

In a clinical setting, data on intralesional neoadjuvant treatments are derived from trials on early metastatic disease (unresectable stage IIIB/C-IVM1a). The combined intralesional administration of Interleukin 2 (IL2) and tumor necrosis factor-alpha (TNFα), both fused with monoclonal antibody L19, which binds fibronectin (immunocytokine L19-IL2/L19TNF), and achieved a disease control rate of 80%. This antitumoral response allowed the surgical resection of residual tumor mass in 7/20 patients [62]. In the OPTiM trial, an RR of 40.5% was reported under an intratumoral injection of the oncolytic virus, Talimogene laherparepvec (T-VEC), in stage IIIB-C and IV M1a melanoma patients [63]. A preliminary analysis of the first T-VEC neoadjuvant trial showed an R0 resection rate of 42.1% versus 37.1% with surgery alone [64]. Both intralesional immunocytokines and oncolytic viruses, alone or in combination with systemic immunotherapy, are now under investigation in a neoadjuvant setting in large studies (NCT03567889, NCT04330430, and NCT03842943).

## 7. Ongoing Clinical Trials

To map the current trends in trial design for melanoma neoadjuvant treatments, we searched and manually annotated the ongoing studies available on clinicaltrial.gov (Table 2 and Table S1 for the complete version; see the Supplementary document Table S2 for the searching method). We included only trials conducted in a neoadjuvant setting and focused on cutaneous resectable melanoma. We excluded studies that allowed the recruitment of multiple solid tumor types.

Our research results focused on 23 trials being considered for review. Before outlining specific studies with probable clinical applications, some general comments can be made. Firstly, the trials were mostly (83%) phase 2 studies. Indeed, the median number of patients planned to be enrolled was medium-to-low (45, interquartile range 29–72). More than half of the trials used the RR (57%) as a primary endpoint, while nearly one third used RFS (30%). A response rate endpoint facilitates rapid data generation, and the surrogacy of melanoma with RFS seems to be probable [20]. Therefore, besides RR, longer endpoints, such as RFS or OS, have been appropriated for neoadjuvant trials. Regarding the drugs used, immunotherapy accounts for 79% of the studies’ treatments. The most common schemes include single (already approved) checkpoint inhibitors (17%), a second-generation immunotherapeutic compound added to a checkpoint inhibitor (17%), and an oncolytic virus added to a checkpoint inhibitor (15%). The BRAF/MEK inhibitors account for one quarter of the trials (15% BRAF/MEK inhibitor alone and 8% BRAF/MEK inhibitors associated with checkpoint inhibitors). The pathways targeted by second-generation immunotherapeutic compounds include interleukin 2 and TNFalpha (Daromun), TIGIT (MK-7684), LAG3 (Relatlimab), TIM3 (TSR-022), TLR9 (CMP-001), and Semaphorins (Pepinemab).

Interestingly, 70% of neoadjuvant trials also scheduled adjuvant treatment. This is particularly important when RFS is considered. Moreover, the intensity of adjuvant treatment could be modulated by the response to neoadjuvant therapy. However, this adjuvant escalation vs. de-escalation based on pCR is being evaluated by only a minority of studies (PRADO, NCT04310397). In general, the overall time of the therapy (neoadjuvant + adjuvant) among trials was 1 year, while the median neoadjuvant length was 6.5 weeks (an interquartile range of 6–8 weeks).

An ongoing randomized phase 2 trial that could be practice changing (NCT03698019) will test 9 weeks of pembrolizumab followed by surgery versus surgery alone. Patients of each arm will undergo adjuvant pembrolizumab to complete 1 year of therapy. The primary endpoint is relapse-free survival, and the target accrual is 500 patients. This study could show whether neoadjuvant immunotherapy can decrease melanoma relapse compared to a classic adjuvant approach.

Another interesting trial is the OpACIN-neo/PRADO study. This is an open-label three-arm phase 2 trial (OpACIN-neo) that tests the different toxicity levels of three Nivolumab and Ipilimumab combinations. The results have already been published (see above). The expansion cohort (PRADO) will enroll 110 stage III melanoma patients who will be treated with the winning treatment of the OpACIN-neo study. Moreover, the adjuvant treatment will be tailored to the neoadjuvant treatment via the achievements of pCR.

## 8. Guidelines for Neoadjuvant Trials (International Neoadjuvant Melanoma Consortium)

**Figure 1:**
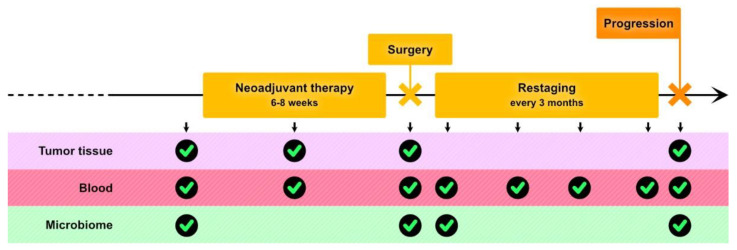
General outline and biospecimen collection for a neoadjuvant trial for melanoma. Adapted from the International Melanoma Neoadjuvant Consortium [65].

To harmonize the study design of neoadjuvant trials on melanoma, in 2016, the International Neoadjuvant Melanoma Consortium was established, and has produced some suggestions for clinical trial development [65]. A focused overview of these key points will be reviewed here (Figure 1).

Stage III is an appropriate setting to conduct a neoadjuvant trial, because it necessitates risk-reduction therapy and allows us to study not only the tumor, but also the lymph nodes after a lymphadenectomy. In that context, stage II or IIIA should have a different and dedicated study design. Instead, oligometastatic stage IV could be included in stage III trials, but in a different cohort, because of its different risk of relapse.

The optimal duration of neoadjuvant therapy remains unknown. However, it is critical to balance efficacy with the risk of progression or the delay of surgery because of toxicity. From this perspective, a duration of 6–8 weeks is suggested. Such a homogenous duration could also permit a cross-trial comparison. However, as new evidence regarding neoadjuvant therapy emerges, a longer duration could be considered.

Besides the neoadjuvant regimen, it is also vital to schedule the adjuvant phase. In light of the data generated from registration phase III adjuvant trials, a total of 1 year of therapy should be considered adequate. However, as new data become available, a tailored adjuvant approach based on the achievement of pCR could be adopted.

Regarding outcomes, several considerations can be made. Compared to stage IV, which is often characterized by immunoevasion [66], earlier stages could generate a strong immune response to neoadjuvant therapy. This could translate into greater toxicity. Therefore, a careful annotation of adverse events or delays in surgery is crucial. RR should be one of the main endpoints. While radiologic RR provides useful information, pathologic RR was shown to be superior [49]. Moreover, in a pooled analysis of four neoadjuvant trials [20], the pathologic response was correlated to improved RFS and OS. However, in melanoma, pCR has yet to be accepted as a surrogate endpoint for survival by regulatory bodies. For this reason, RFS, melanoma-specific survival, and OS should be collected. Moreover, the annotation of local- and distant-relapse could help track virtually curable non-responders.

Basal staging imaging should be provided for each candidate in neoadjuvant trials. While 18F-FDG PET/CT offers high sensitivity, a CT scan is preferred, because of its better visualization of the liver, brain, and lungs.

From a translational point of view, a careful and pre-planned collection of biological samples should be done. Cancer cells and their microenvironment, blood (for circulating tumor DNA, exosomes, and other circulating molecules), and the microbiome should be collected at specific time points (Figure 1).

Another possible innovative strategy to reduce systemic toxicity and further improve the tumoral delivery of drugs could be intratumoral immunotherapy, as stated in a recent ESMO-meeting [67].

Finally, it should be stated that current international guidelines do not support neoadjuvant therapies for resectable disease outside the frame of clinical trials.

## 9. Future Directions

It has been hypothesized that neoadjuvant treatments could be more effective compared to adjuvant ones. This idea relies on the preclinical and clinical findings that neoadjuvant immunotherapy sharply increases the number of TILs [21], which are related to a better prognosis [68]. Moreover, in the OpACIN trial discussed previously, the RFS was more significantly improved in the neoadjuvant arm compared to the adjuvant one [22]. However, since this trial was not powered to show differences in the RFS between the two arms, a formal demonstration of the clinical superiority of neoadjuvant therapy is still lacking. Some ongoing clinical trials (e.g., NCT03698019) could offer more guidance for the future.

Another unresolved issue is the proper duration of neoadjuvant therapy. While clear data regarding the optimal duration of targeted therapy are still lacking, the use of only two cycles of Ipilimumab + Nivolumab seems to be effective in a neoadjuvant setting [52]. These findings also agree with recent data presented at ASCO 2020 for a metastatic setting [69]. Therefore, two cycles of Nivolumab (3 mg/kg) and Ipilimumab (1 mg/kg) could provide a reasonable starting point for future clinical trials. This combination seems to offer the best efficacy/toxicity ratio [51]. From this perspective, significant efforts should be made to identify the predictive biomarkers of pCR to design escalation trials in putative non-responder patients. The tumor mutational burden [68], TILs [68], and gene-expression signatures [68] could be used individually or together to better predict pCR.

Finally, an important unresolved question is how to tailor adjuvant treatments after neoadjuvant therapy. Since pCR seems to predict an excellent prognosis, active surveillance could be sufficient for complete responders. On the contrary, patients that do not obtain a pathological response to neoadjuvant treatment could benefit from an escalated adjuvant approach. These ideas are being tested in the ongoing PRADO trial discussed previously [70].

## 10. Conclusions

The pharmacological treatment of melanoma is a rapidly evolving field. Adjuvant immunotherapy and BRAF/MEK inhibitors have revolutionized the prognosis of this highly lethal disease, and the neoadjuvant approach promises a second revolution. Pre-surgical therapy could exploit a competent immune system to increase the efficacy of currently available drugs, while providing important biologic and translational data. Moreover, the achievement of pCR could help guide a more tailored adjuvant approach. While neoadjuvant therapy for stage III melanoma is an exciting topic for the future, some challenges still need to be addressed, such as the high toxicity of such therapies and determining the most effective schemes and schedules. Ongoing clinical trials will provide all these awaited data.

## Figures and Tables

**Table 1:** Key neoadjuvant trials for stage III melanoma.

Study	Start	Completion	Neoadjuvant Drugs	Adjuvant Drugs	N	pCR	RFS (mo)	AE ≥3	Biomarkers
OpACIN-neo NCT02977052	2016	Ongoing (recruiting)	Arm A: Ipi3 + Nivo1 × 2 cycles Arm B: Ipi1 + Nivo3 × 2 cycles Arm C: Ipi3 × 2 cycles followed by Nivo3 × 2 cycles	-	Arm A: 30 Arm B: 30 Arm C: 26	Arm A 47% Arm B 57% Arm C 23% Near-pCR Arm A 23% Arm B 7% Arm C 23%	24-mo RFS: Arm A: 90% Arm B: 77% Arm C: 83%	Arm A: 40% Arm B: 20% Arm C: 50%	PD-L1 expression did not influence pathologic response rate Baseline high IFN-γ signature and TMB were associated with pathologic response and lower risk of relapse Lower baseline intestinal microbial diversity in non-responders
OpaCIN NCT02437279	2015	n/a	Arm A: - Arm B: Ipi3 + Nivo1 × 2 cycles	Arm A: Ipi3 + Nivo1 × 4 cycles Arm B: Ipi3 + Nivo1 × 2 cycles	Arm A: 10 Arm B: 10	33%	30-mo RFS: Arm A: 60% Arm B: 90%	Arm A: 90% Arm B: 90%	Greater expansion of T cell clones in neoadj arm as compared to adj treatment Higher number of newly detected T clones in non-relapsed patients Low CD3, beta2 microglobulin, PDL-1 expression and IFN-gamma signature were associated with relapse
Amaria NR et al. NCT02519322	2016	Ongoing (recruiting)	Arm A: Nivo3 x4 cycles Arm B: Ipi3 + Nivo1 × 3 cycles	Nivo3 up to 13 cycles	Arm A: 12 Arm B: 11	Arm A: 25% Arm B: 45%	20-mo RFS: Arm A: 56% Arm B 81%	Arm A: 8% Arm B: 73% No G4/5 in any group	Higher total TMB associated with response Higher pre-existing T-cell clonality in responders
Huang et al. NCT02434354	2015	Ongoing (recruiting)	Pembrolizumab (200mg) 1 dose	Pembrolizumab up to 1 year	29	19% near-pCR: 11%	24-mo RFS: 63%	21%	1-yr RFS 89% in brisk TILs vs 1-yr RFS 27% in non-brisk TILs Enrichment of CD39+ T-cells subset related to response to PD1 blockade Eomes T-bet Tex were associated with clinical benefit
Tarhini et al. NCT00972933	2009	2013	Ipi10 × 2 cycles	Ipi10 × 2 cycles	35	0%, near-pCR: 15%	Median PFS 10.8 mo	Diarrhea/colitis 14%, hepatitis 6%, rash 3%, elevated lipase 9%	Association between lower baseline Treg and PFS Decreased MDSC were associated with improved PFS Baseline IL-17 level significantly associated with severe colitis/diarrhea Baseline TGF-beta and IL-10 level were prognostic
Combi-Neo NCT02231775	2014	Ongoing (recruiting)	Dabrafenib + Trametinib × 8 wk	Dabrafenib + Trametinib up to 44 wk	21	58%	Median RFS: 19.7 mo	15%	Lower levels of pERK in responders Upregulation of cytotoxic CD8+ T-cell transcripts in pts with pCR
NeoCombi NCT01972347	2014	Ongoing (not recruiting)	Dabrafenib + Trametinib × 12 wk	Dabrafenib + Trametinib up to 40 wk	35	49%	Median RFS: 23.3 mo	29%	Higher proportion of PD-L1-positive and SOX10-positive cells in pts with pCR Higher density of intratumoural CD8-positive T cells in pCR pts Higher expression of TIM3 and LAG3 on T-cells of non-pCR pts
Buzaid AC et al. (1998)	n/a	n/a	Cisplatin, Dacarbazine, Vinblastine, IFN, IL-2 × 2-4 cycles	Cisplatin, Dacarbazine, Vinblastine, IFN, IL-2	62	6.5%	-	-	-
Gibbs P et al. (2002)	n/a	n/a	Cisplatin, Dacarbazine, Vinblastine, IFN, IL-2 × 2 cycles	Cisplatin, Dacarbazine, Vinblastine, IFN, IL-2	48	11.1%	-	Hypotension 100%, neutropenia 56%, thrombocytopenia 56%	-
Andbacka RH et al. NCT02211131	2015	Ongoing (not recruiting)	T-VEC	-	194	15.8%	-	-	-

AE = adverse events, Ipi = Ipilimumab, Ipi1 = Ipilimumab 1 mg/kg, Ipi3 = Ipilimumab 3 mg/kg, mo = months, Nivo = Nivolumab, Nivo1 = Nivolumab 1 mg/kg, Nivo3 = Nivolumab 3 mg/kgRFS = recurrence-free survival, RR = response rate, RT = radiotherapy, SNL = sentinel lymph node, TILs = Tumor-infiltrating lymphocytes, TMB = tumor mutational burden, yr = year, wk = week. The data were collected from the published works and/or from http://clinicaltrials.gov. See the text for references.

**Table 2:** Ongoing neoadjuvant trials for stage III melanoma.

Study	Neoadjuvant Drugs	Adjuvant Drugs	N	Primary Outcome
NCT04020809	Atezolizumab for 6 weeks	-	20	Feasibility, safety
NCT02036086	Vemurafenib + Cobimetinib for 8 weeks	Vemurafenib + Cobimentinib to complete 1 year of therapy	24	Feasibility
NCT04197882	OrienX010 + Treprizumab for 8 weeks	Treprizumab up to 1 year	30	pCR, RR
NCT03757689	Pembrolizumab single dose	Pembrolizumab up to 1 year	63	SLN positivity, safety
NCT04207086 - Neo PeLe	Pembrolizumab + Lenvatinib for 6 weeks	Pembrolizumab to complete 1 year of therapy	20	RR, immune response
NCT04330430	T-VEC + Nivolumab for 8 weeks	-	24	RR
NCT02211131	T-VEC + surgery vs. surgery	-	150	RFS
NCT04133948	Nivolumab vs. Nivolumab + Domatinostat vs. Nivolumab + Domatinostat + Ipilimumab for 6 weeks	-	45	Feasibility
NCT04248387	Toripalimab for 4 weeks	-	100	pCR
NCT03567889	Daromun for 4 weeks + surgery vs. surgery	-	248	RFS
NCT03554083	Cobimetinib + Atezolizumab ± Vemurafenib for 12 weeks	Atezolizumab for 6 months	30	pCR, RFS
NCT02858921 - NeoTrio	Sequential vs. concurrent Dabrafenib + Trambetinib + Pembrolizumab vs. Pembrolizumab for 6 weeks	Pembrolizumab to complete 1 year of therapy	60	RR
NCT03842943	T-VEC + Pembrolizumab for 6 months	Pembrolizumab up to 1 year	28	pCR
NCT04303169	Pembrolizumab + MK-7684 vs. Pembrolizumab + V937 vs. Pembrolizumab	Pembrolizumab to complete 1 year of therapy	65	Feasibility, safety, pCR
NCT02977052 - OpACIN-neo/PRADO	Ipilimumab 1 mg/kg + Nivolumab 3 mg/kg for 4 weeks	Observation (if pCR) vs. CLND (if pRP) vs. CLND + RT + Nivolumab to complete 1 year of therapy	110	Safety, RR, pCR, RFS
NCT04310397	Dabrafenib + Trametinib for 8 weeks	Dabrafenib + Trametinib ± Spartalizumab (if no pCR) to complete 1 year of therapy	45	RFS
NCT03698019	Pembrolizumab for 9 weeks + surgery vs. surgery	Pembrolizumab to complete 1 year of therapy	500	RFS
NCT04221438	Encorafenib + Binimetinib for 8 weeks	Encorafenib + Binimetinib for 11 months	42	pCR, PET/TC changes
NCT04139902	Dostarlimab for 6 weeks vs. Dostarlimab + TSR-022 for 6 weeks	Dostarlimab to complete 1 year of therapy	56	RR
NCT03618641	Nivolumab + CMP-001 for 7 weeks	Nivolumab + CMP-001 to complete 1 year of therapy	32	RR
NCT02231775	Dabrafenib + Trametinib for 8 weeks	Dabrafenib + Trametinib to complete 1 year of therapy	78	RFS
NCT01321437	Axitinib for 8 weeks	Axitinib until progression or unacceptable toxicity	11	RR
NCT03769155	Pepinemab ± Nivolumab ± Ipilimumab	-	36	TILs
Eudract: 2018-002172-40	Ipilimumab 1 mg/kg + Nivolumab 3 mg/kg for 12 weeks	Nivolumab 480 mg for 6 months	n/a	pCR

CLND = complete lymph node dissection, RFS = recurrence-free survival, RR = response rate, RT = radiotherapy, SNL = sentinel lymph node, TILs = Tumor-infiltrating lymphocytes. The data were collected from clinicaltrials.gov.

## Supplementary Materials

The following are available online at https://www.mdpi.com/2072-6694/12/7/1941/s1, Table S1: Ongoing neoadjuvant clinical trials in melanoma, Table S2: Search query on clinicaltrial.gov: melanoma.

## Abbreviations

CR	complete responses
CTCs	circulating tumor cells
DMFS	distant metastases-free survival
ICIs	immune checkpoint inhibitors
IL-2	Interleukin-2
INF-alpha	Interferon-alpha
IPI	ipilimumab
irAEs	immune-related adverse events
NIVO	nivolumab
OS	overall survival
pCR	pathologic complete response
pRR	pathologic response rate
RFS	relapse-free survival
RR	response rate
TILs	tumor-infiltrating lymphocytes
TMB	tumor mutational burden
Treg	T-regulatory cells
