# Supplementary material for: “To Anticipate”: Neoadjuvant Therapy in Melanoma with a Focus on Predictive Biomarkers
Source: Cancers (Basel). 2020 Jul 17;12(7):1941. doi: 10.3390/cancers12071941 (PMC7409214; doi:10.3390/cancers12071941)
Supplement: Supplementary file 1 [file cancers-12-01941-s001.zip › supporting information/Table S2 Search query on clinicaltrial.gov melanoma.docx]

**Table S2.** Search query on clinicaltrial.gov: melanoma.

| **Search Query on Clinicaltrial.gov: Melanoma.** | |
| --- | --- |
| - Inclusion criteria: - Interventional trials - Phase 1 early, phase 1, phase 2, or phase 3 trials - Not yet recruiting, recruiting, enrolling by invitation, or active, not recruiting trials - Stage I-IV (oligometastatic and resectable) melanoma - Treatment-naïve melanoma | - Exclusion criteria: - Clinicaltrial.gov trial results available - Trial results already published - Other neoplasm included in the study - Unresectable stage III-IV melanoma |
